# Effects of time-restricted eating on weight loss, sleep, and quality of life in obstructive sleep apnea-hypopnea syndrome (OSAHS) patients with obesity: a randomized controlled trial

**DOI:** 10.3389/fnut.2026.1739995

**Published:** 2026-02-17

**Authors:** Yi Wen, Xia Yang, Jinglan Chen, Shiqi Xie, Jianrong Zhou, Xiaozhu Zhang

**Affiliations:** 1 School of Nursing, Chongqing Medical University, Chongqing, China; 2 School of Social Development and Public Policy, Fudan University, Shanghai, China; 3 Department of Eye and Otorhinolaryngology, Women and Children's Hospital of Chongqing Medical University, Chongqing, China; 4 Department of Otorhinolaryngology, The First Affiliated Hospital of Chongqing Medical University, Chongqing, China

**Keywords:** obstructive sleep apnea-hypopnea syndrome, obesity, time-restricted eating, sleep quality, quality of life, randomized controlled trial

## Abstract

**Background:**

Weight loss is a key adjunctive therapy for obstructive sleep apnea-hypopnea syndrome (OSAHS) patients with obesity; however, its long-term success rate remains low. Time-restricted eating (TRE) is an emerging dietary strategy for weight-loss, but its application in obese OSAHS patients is still limited. This study aimed to evaluate the effects of TRE on weight loss, sleep, and quality of life (QoL) in obese OSAHS patients.

**Methods:**

In this randomized controlled trial, 68 obese adults with OSAHS were allocated (1:1) to an 8-h TRE group (eating window: 10 a.m. to 6 p.m.) or a control group receiving standard care for 12 weeks. The primary outcomes were changes in body mass index (BMI) and sleep quality (Pittsburgh Sleep Quality Index, PSQI). Secondary outcomes included changes in daytime sleepiness (Epworth Sleepiness Scale, ESS), QoL (Calgary Sleep Apnea Quality of Life Index, SAQLI), and OSAHS risk (STOP-Bang questionnaire, SBQ).

**Results:**

Sixty participants completed the study. At 12 weeks, while the between-group difference in BMI change was not significant [adjusted mean difference (MD): −0.18 kg/m^2^; *p* = 0.641], the TRE group showed a significant within-group reduction (−0.86 kg/m^2^; *p* = 0.033). Compared with the control group, the TRE group showed greater improvements in sleep latency (adjusted MD: 0.44; *p* = 0.007), sleep duration (adjusted MD: 0.54; *p* = 0.003), and sleep efficiency (adjusted MD: −0.55; *p* = 0.001). The TRE group also had greater improvements in the total QoL score (adjusted MD: 0.36; *p* = 0.027) and in the emotional functioning (adjusted MD: 0.58; *p* = 0.008).

**Conclusion:**

A 12-week 8-h TRE intervention effectively induced weight loss and improved specific aspects of sleep and QoL in obese patients with OSAHS. TRE shows promise as a beneficial dietary intervention within the comprehensive management of OSAHS and obesity.

## 1 Introduction

Obstructive sleep apnea-hypopnea syndrome (OSAHS) is a common sleep-related breathing disorder in adults, which is characterized by the recurrent collapse or obstruction of the upper airway during sleep (1, 2). Globally, a recent meta-analysis estimated that approximately 54% of the world’s population is affected by OSAHS (3), with an estimated 936 million adults aged 30 to 69 years worldwide suffering from this disorder (4). In China, the prevalence of OSAHS among adults is 3.93%, with rates of 5.69% in males and 2.17% in females (5). According to statistics, OSAHS influences approximately 176 million people in China (4), with over 60 million requiring active intervention (5). Without timely treatment, the persistent chronic intermittent hypoxia and disrupted sleep architecture experienced by OSAHS patients over the long term can increase the risk of associated long-term health complications (6–8). Importantly, un-treated OSAHS is also associated with increased mortality, placing significant strain on healthcare systems worldwide (9, 10).

Obesity is one of the most well-established risk factors for the development and progression of OSAHS (11–13). Body mass index (BMI) is widely utilized as a predictor for OSAHS severity (14–16). Among adults with a BMI ≥ 30 kg/m^2^, the prevalence of OSAHS is approximately 40% (11). Progressive weight gain has been shown to lead to a persistent increase in the apnea-hypopnea index (AHI), resulting in the worsening of OSAHS (17–19). In contrast, the increased appetite and altered energy metabolism caused by OSAHS may maintain or even exacerbate obesity (20, 21). Therefore, the relationship between OSAHS and obesity is not a simple one-way causality, but rather a complex, bidirectional interaction characterized by a vicious cycle of mutual aggravation. The co-existence of OSAHS and obesity has a profound and multifaceted consequences on patients’ health, involving numerous different systems and functions, including the cardiovascular system (22–24), metabolic disorders (25), and cognitive decline (26, 27). Furthermore, the concurrence of OSAHS and obesity creates a compounding effect of nocturnal sleep apnea, excessive daytime sleepiness, fatigue, and mood disorders, which are detrimental to both sleep quality and overall quality of life (28).

Continuous positive airway pressure (CPAP) therapy is widely recognized as the gold standard treatment for patients with OSAHS (9, 29). However, CPAP therapy is often associated with issues such as poor patient tolerance and compliance, maintenance difficulties, and limited portability (30, 31). Additionally, potential adverse reactions such as skin allergies, nasal congestion, airway dryness, or claustrophobia contribute to low long-term adherence rates (9, 32). In OSAHS patients with obesity, CPAP therapy does not resolve the underlying pathophysiological mechanisms. Weight reduction represents an important complementary therapy for this patient population (11, 33). Previous randomized clinical trials have reported that a decrease in BMI is positively correlated with the severity of OSAHS (34–36). Although OSAHS management guidelines from various health organizations emphasize the importance of weight loss for obese individuals with OSAHS, achieving successful and sustained weight reduction through conventional interventions such as long-term caloric restriction and regular exercise remains challenging.

Recently, time-restricted eating (TRE) has emerged as an increasingly popular nutritional strategy for weight loss, allowing unrestricted food intake within a limited 4 to 10 h per day eating window, followed by fasting for the remaining 14 to 20 h (37, 38). TRE does not impose mandatory daily calorie restrictions, making it a potentially sustainable dietary management strategy for individuals who struggle to adhere to conventional energy-restricted diets (39). Previous studies have shown that TRE facilitates weight loss in obese individuals by unconsciously reducing caloric intake and improving metabolic homeostasis (40–42). Additionally, time-restricted eating may enhance sleep quality by aligning circadian rhythms and reducing late-night eating behavior, ultimately leading to an overall improvement in sleep and quality of life (43).

Although weight loss is associated with improved sleep and quality of life in obese individuals, studies focusing on OSAHS patients with obesity remain limited. Moreover, research on the application of time-restricted eating in OSAHS patients with obesity remains scarce to date, and the impact of TRE on core clinical outcomes such as sleep quality and quality of life has been largely neglected. Therefore, this study aims to explore the effects of time-restricted eating on weight loss, sleep quality, and quality of life in OSAHS patients with obesity, thereby providing a reference for the development of novel integrated intervention strategies in future research. We hypothesized that, compared with the control group, patients in the TRE group would result in greater weight loss after 12 weeks of intervention and show sustained improvements in sleep quality and quality of life.

## 2 Materials and methods

### 2.1 Study design

This study was a parallel-group, randomized controlled trial conducted over a period of 12 weeks. Participants were randomly assigned to the time-restricted eating intervention group and the control group. All outcomes were assessed at baseline and at the end of the 12-week intervention. This study was approved by the Ethics Committee of the First Affiliated Hospital of Chongqing Medical University (Approval No. 2022–052) and registered with the Chinese Clinical Trial Registry (Registration No. ChiCTR2200060631). This study was conducted in accordance with the principles of the Declaration of Helsinki and adhered to the Consolidated Standards of Reporting Trials (CONSORT) reporting guideline for RCTs. All participants provided written informed consent.

### 2.2 Participants

Participants were recruited from the sleep laboratory of the otolaryngology out-patient department at a tertiary hospital in Chongqing, China. The inclusion criteria were: (1) participants with mild or higher severity of OSAHS (AHI ≥ 5 events/h); (2) BMI of 28–45 kg/m^2^; (3) age 18–60 years; (4) ability to use a smartphone or mobile device; (5) willingness to participate in this study and provide written informed consent. Exclusion criteria were as follows: (1) current or planned treatment with either surgery or CPAP for OSAHS; (2) participants with severe cardiopulmonary disease, hepatic or renal dysfunction, malignant tumors, other serious organ diseases, or those currently taking weight-loss medications; (3) individuals with psychiatric disorders or cognitive impairment; (4) those who were pregnant or planning pregnancy. The withdrawal criteria included: (1) voluntary withdrawal from the study; (2) lost to follow-up for various reasons.

### 2.3 Randomization

Participant recruitment and randomization took place from April to December 2022, with allocation to the TRE group or the control group at a 1:1 ratio. A statistician not involved in the study used a computer to generate a random number sequence, which was then printed on allocation cards and placed in sealed, opaque envelopes. Another researcher subsequently opened the envelopes according to participant enrollment order, and participants were assigned to either the TRE group or the control group based on the instructions inside the respective envelope.

### 2.4 Intervention

The TRE intervention was systematically developed using the Behavior Change Wheel (BCW) framework, a comprehensive model for designing behavior change interventions (44). The process involved three core stages: (1) Understanding the Behavior: The target behavior was defined as “adhering to an 8-hour eating window daily.” Behavioral analysis using the COM-B model (Capability, Opportunity, Motivation-Behavior) (44) and the Theoretical Domains Framework (TDF) (45) identified key determinants to address. (2) Identifying Intervention Options: Based on pre-trial structured interviews with obese OSAHS patients, key behavioral determinants were identified, guiding the selection of intervention functions: education, incentivization, environmental restructuring, and training. (3) Identifying Content and Implementation Options: Specific Behavior Change Techniques (BCTs) were selected corresponding to the chosen intervention functions (46). These BCTs included, but were not limited to, information about health benefits, instruction on how to perform the behavior, self-monitoring of behavior, feedback on behavior, social support (practical), and review of behavior goals.

Participants in the TRE group were instructed to adhere to an 8-h eating window (10:00 a.m. to 6:00 p.m.). During the eating window, participants could consume food in any quantity and of any composition. Outside the window, calorie intake was prohibited, with the stipulation that only water and energy-free beverages like unsweetened coffee and tea were permitted. Participants were instructed to maintain the same eating window throughout the 12-week intervention period. Adherence was monitored using standardized paper forms where participants recorded their first and last eating times each day. Researchers reviewed these records every 4 weeks. Adherence was defined as confining intake to the 8-h window for at least 6 days per week. To reduce participant burden and enhance compliance, detailed food logging was not required. Additionally, participants received guidance on physical activity recommendations outlined in obesity prevention and management guidelines during the 12-week study period.

Besides standard care of OSAHS management, the specific intervention measures for the TRE group were developed using the BCW guidebook. All intervention components were delivered through face-to-face consultation, telephone call, and online platform. The primary components included face-to-face in-person educational sessions delivered by trained intervention researchers to participants. These sessions covered topics such as the health risks associated with OSAHS and obesity, establishing accurate disease awareness, correcting patient misconceptions, and teaching participants how to calculate their BMI and understand the normal range for self-monitoring purposes. Additionally, participants were informed about the fundamental principles and health benefits of time-restricted eating and advised to set periodic weight loss goals. Researchers established a peer support group based on online platform to foster communication and mutual encouragement between participants. Every 4 weeks, researchers conducted online support meetings to document adverse events, review participants’ weight loss progress milestones, and provide solutions to emerging issues. Furthermore, participants collaborated with researchers to develop weight management plans and goals for the next phase.

During the 12-week study period, participants in the control group were offered standard care protocols from the sleep laboratory. This included basic OSAHS health education and general nutritional counseling aligned with the Chinese Dietary Guidelines. The nutritional counseling advised a balanced diet without specific caloric limits or personalized meal plans, emphasizing increased intake of vegetables, fruits, whole grains, and lean protein, while reducing consumption of cooking oil, added sugars, and ultra-processed foods. Education also highlighted regular physical activity, proper sleeping positions, smoking cessation, and alcohol moderation. No specific caloric targets or daily food intake monitoring was provided. After the study, participants in the control group were offered an introduction to the intervention program and obtain electronic materials.

### 2.5 Outcome measures

All outcomes were measured at both baseline and Week 12. Primary outcomes were BMI and sleep quality. Secondary outcomes included changes in excessive daytime sleepiness, quality of life, and the change in STOP-Bang questionnaire score. Furthermore, sociodemographic information and disease indicators were collected at baseline, including age, gender, educational level, occupational status, marital status, family monthly household income, and AHI. As a process measure, adherence to the TRE protocol was calculated as the percentage of days where self-reported caloric intake was confined to the 8-h window.

The calculation of BMI used a standard formula (weight in kilograms divided by height in meters squared). Participants were asked to record their weight in the morning upon waking, barefoot and wearing light clothing.

Sleep quality change was measured with the Pittsburgh Sleep Quality Index (PSQI) (47). This scale comprises seven components, including subjective sleep quality, sleep latency, sleep duration, sleep efficiency, sleep disturbances, sleep medicine use, and daytime dysfunction. Each component is scored on a scale of 0 to 3, and the sum of the scores for each dimension constitutes the PSQI total score. The total score ranges from 0 to 21 points, with higher scores indicating poorer sleep quality.

The STOP-Bang questionnaire (SBQ) was utilized to assess the severity of OSAHS (48). This instrument has been validated in Chinese populations and demonstrates good diagnostic performance for OSAHS (49). It consists of 8 items, yielding a total score of 0–8. A score of ≥3 indicates a high risk of moderate to severe OSAHS.

The Epworth Sleeping Scale (ESS) is an 8-item instrument designed to evaluate excessive daytime sleepiness in patients (50). It has also been validated and is frequently employed in patients with OSAHS (51). Excessive daytime sleepiness was selected as a secondary outcome because it serves as a key clinical marker of OSAHS severity and directly impacts quality of life (52). Measuring changes in ESS scores allowed to explore whether TRE could alleviate this specific symptom beyond weight loss. The total score ranges from 0 to 24, with higher scores indicating more severe daytime sleepiness.

The Calgary Sleep Apnea Quality of Life Index (SAQLI) was primarily used to measure the quality of life in adult patients with OSAHS (53). This instrument consists of 35 items, grouped into 5 subscales: daily functioning, social interactions, emotional functioning, symptoms, and treatment-related symptoms. Since none of the participants in this study had undergone any treatment, only the first four subscales were involved. The total score was the average of four subscales, ranging from 1 to 7 points, with higher scores indicating better quality of life.

### 2.6 Statistical analysis

For the sample size calculation, we employed a two-sample mean comparison superiority trial approach for estimation. Based on data from a prior report suggesting the efficacy of similar interventions for weight loss (54), we anticipated that the TRE group would achieve a 5% greater reduction in BMI compared to the control group after 12 weeks. With a type-I error rate of 0.05, an estimated sample size of 37 participants per group would provide at least 80% power at week 12 to detect a statistically significant difference in BMI between the TRE group and the control group. Regarding the 10% dropout rate, the final total sample size was 82 participants (41 per group). However, due to recruitment constraints imposed by the COVID-19 pandemic, the final sample size was limited to 68 participants. A post-hoc power analysis using G*Power software, based on the final sample size (*n* = 60), an observed effect size (Cohen’s d) of approximately 0.35 for the primary outcome (BMI), and *α* = 0.05, indicated a statistical power of approximately 58%, which is below the conventional 80% threshold. To address methodological limitation, the trial design, statistical analysis plan, and this specific issue were reviewed by an independent statistician. It was acknowledged that the reduced power limits the definitive conclusions; however, the data are valid for estimating effect sizes and generating hypotheses. This limitation is explicitly considered in the interpretation of the findings.

Statistical analysis was performed using SPSS version 26.0 software (IBM, Chicago, IL, United States). Unless otherwise specified, continuous variables were presented as mean ± standard deviation (SD), while categorical variables were presented as frequencies (percentages). Differences between the two baseline groups were analyzed using *t*-tests for continuous variables or Chi-squared tests for categorical variables. For within-group comparisons, paired sample *t*-tests or Wilcoxon signed-rank tests were used to analyze the differences before and after the intervention, respectively. The primary analysis for between-group comparisons over time was conducted using linear mixed models, following the intention-to-treat principle. The models contained fixed effects for group, time, and the group-by-time interaction, with participant included as a random intercept. All models were adjusted for the respective baseline outcome value. To control for potential confounding, the following baseline covariates were also included in all primary models: age, gender, education level, employments status, marital status, family monthly income, and AHI. Additionally, in exploratory analyses, categorical and continuous outcomes were analyzed with the chi-square test and one-way ANOVA, respectively. Associations were expressed as odds ratio (OR) with 95% confidence intervals from logistic regression. A two-sided *p* < 0.05 was considered statistically significant.

## 3 Results

### 3.1 Participants characteristics

**Figure 1:**
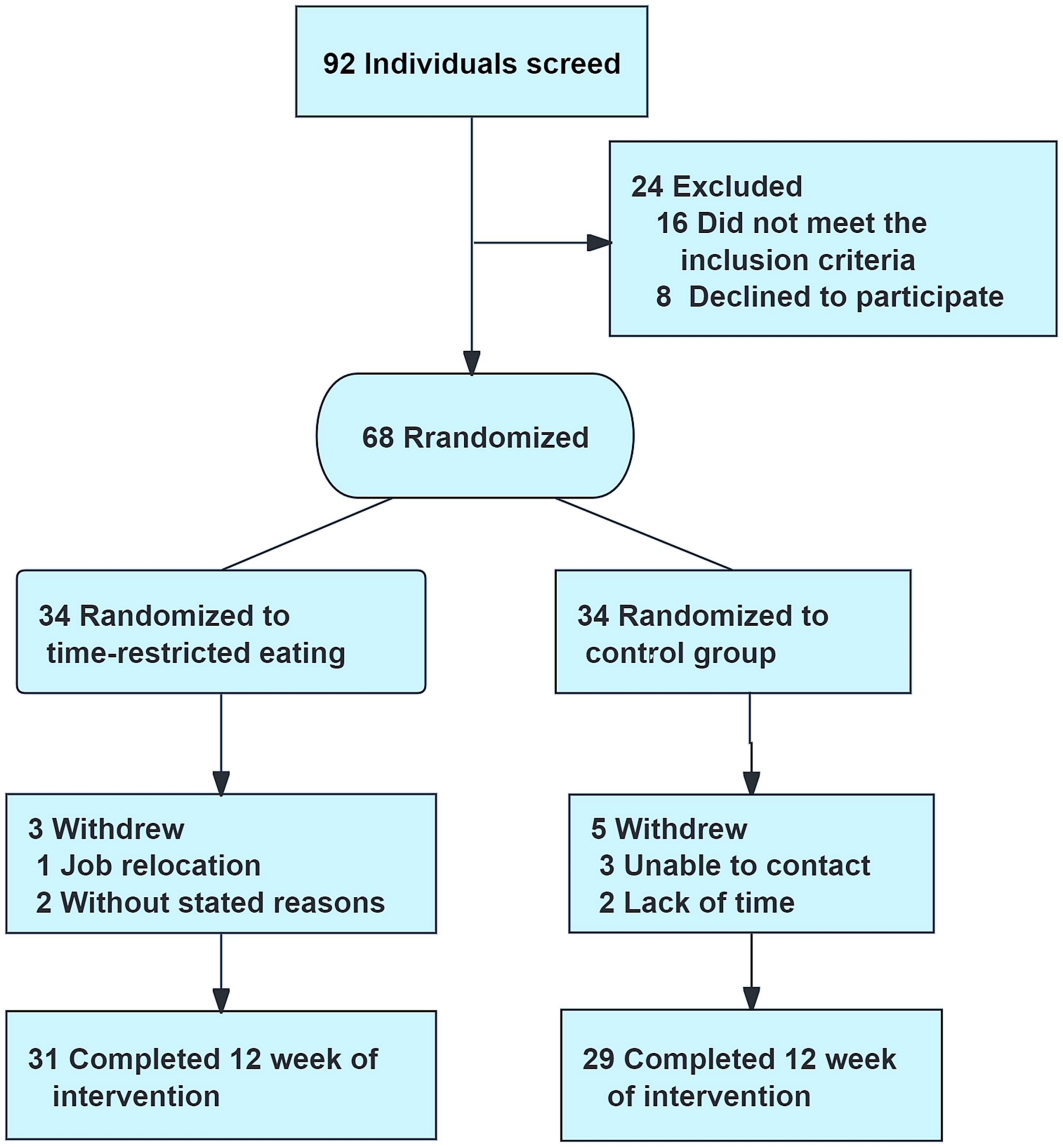
Flowchart of the study participants.

A total of 92 individuals were screened, and 68 were randomized to the TRE group (*n* = 34) or the control group (*n* = 34). Sixty participants completed the study (Figure 1). Baseline demographic and clinical characteristics were similar between the two groups, as shown in Table 1. Furthermore, scores for outcome measures at baseline were comparable, which are summarized in Table 2.

**Table 1:** Baseline demographic characteristics of participants.

Variables	TRE (*n* = 34) Mean ± SD or *n* (%)	Control (*n* = 34) Mean ± SD or *n* (%)	*t*/*χ*^2^	*p*-value
Age (year)	43.47 ± 9.64	43.53 ± 11.81	−0.022	0.982^a^
Gender			0.770	0.380^b^
Male	28(82.4)	25 (73.5)		
Female	6 (17.6)	9 (26.5)		
BMI (kg/m^2^)	30.77 ± 3.02	30.67 ± 2.53	0.142	0.887^a^
AHI (events/h)	44.28 ± 26.28	49.59 ± 24.30	−0.866	0.390^a^
Education level			4.211	0.122^b^
Primary school or below	4 (11.8)	11 (32.4)		
Secondary education	19 (55.9)	15 (44.1)		
Tertiary education	11 (32.4)	8 (23.5)		
Employment status			0.614	0.878^c^
Full-time	21 (61.8)	20 (58.8)		
Part-time	3 (8.8)	5 (14.7)		
Unemployed	10 (29.4)	9 (9.5)		
Marital status			1.197	1.000^c^
Married	19 (55.9)	20 (58.8)		
Single/divorced/widowed	15 (44.1)	14 (41.2)		
Family monthly income			0.074	0.964^b^
<5,000RMB	17 (50.0)	16 (47.1)		
5,000-8,000RMB	11 (32.4)	12 (35.3)		
>8,000RMB	6 (17.6)	6 (17.6)		

TRE, time-restricted eating group; BMI, body mass index; AHI, apnea-hypopnea index. ^a^ *t*-test; ^b^ chi-squared tests; ^c^ Fisher’s exact test.

**Table 2:** Comparison of baseline outcome measures between the two groups.

Variables	TRE (*n* = 34) Mean ± SD	Control (*n* = 34) Mean ± SD	*t*	*p*-value
PSQI	8.12 ± 2.86	7.94 ± 3.83	0.215	0.830
Subjective sleep quality	1.12 ± 1.07	1.47 ± 0.90	−1.478	0.144
Sleep latency	1.56 ± 0.96	1.44 ± 1.05	0.482	0.631
Sleep duration	0.94 ± 0.95	0.76 ± 0.99	0.751	0.455
Sleep efficiency	0.50 ± 0,93	0.79 ± 1.15	−1.161	0.250
Sleep disturbances	1.65 ± 0.92	1.56 ± 1.05	0.369	0.713
Sleep medication use	0.68 ± 0.91	0.38 ± 0.78	1.430	0.157
Daytime dysfunction	1.68 ± 1.12	1.53 ± 1.11	0.544	0.588
SBQ	4.32 ± 1.17	4.18 ± 1.11	0.530	0.598
ESS	9.91 ± 4.80	8.09 ± 4.80	1.568	0.122
SAQLI	4.07 ± 1.11	3.99 ± 0.86	0.341	0.734
Daily functioning	4.93 ± 1.55	4.86 ± 1.25	0.209	0.835
Social interactions	4.98 ± 1.44	4.87 ± 1.46	0.306	0.760
Emotional functioning	5.40 ± 1.44	4.95 ± 1.27	1.354	0.180
Symptoms	1.16 ± 0.36	1.26 ± 0.35	−1.169	0.247

TRE, time-restricted eating group; PSQI, Pittsburgh Sleep Quality Index; SBQ, STOP-Bang questionnaire; ESS, Epworth Sleeping Scale; SAQLI, Calgary Sleep Apnea Quality of Life Index. *p*-value, *t*-test for continuous variables.

### 3.2 Weight loss

**Figure 2:**
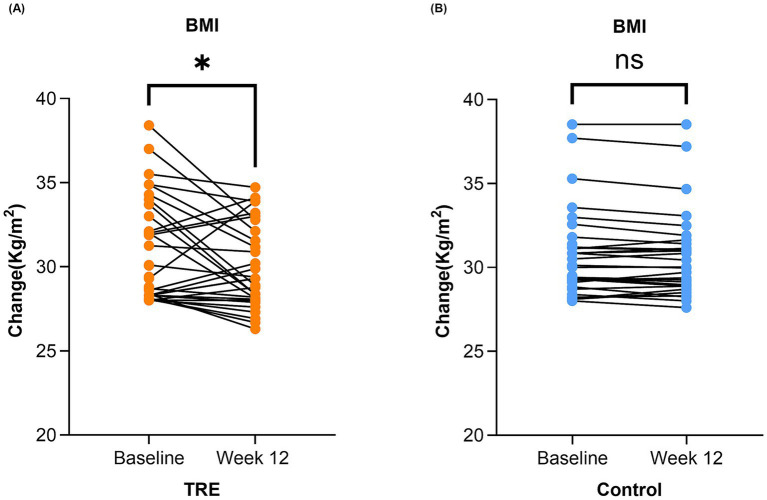
Change in BMI in the TRE and control group after 12 weeks. BMI, body mass index. TRE, time-restricted eating group. **(A)** Change from baseline to week 12 in BMI in the TRE group. Paired sample *t*-test was used. **(B)** Change in BMI in the control group from baseline to week 12. Paired sample *t*-test was used.

Linear mixed model analysis found no significant effect of TRE on BMI change over time [F (1,128) = 1.56; *p* = 0.214]. The between-group comparison showed no statistically significant difference in BMI change at 12 weeks (adjusted mean difference [MD]: −0.18 kg/m^2^, 95% CI: −0.96 to 0.59; *p* = 0.641; Table 3). However, a distinct pattern emerged from the within-group analyses. Intra-group analysis revealed a significant reduction in BMI from baseline within the TRE group (−0.86 ± 0.55 kg/m^2^; *p* = 0.033; Figure 2A), with a mean (SD) adherence of 5.2 (0.7) days per week (74.8% of days over 12 weeks) to the eating window. In contrast, no significant change was observed within the control group (0.02 ± 0.05 kg/m^2^; *p* = 0.125, Figure 2B).

**Table 3:** Change in BMI, SBQ, ESS, sleep and quality of life by week 12.

Variables	Change from baseline to week 12				Adjusted mean difference between groups at week 12 (95%CI)	
	TRE (*n* = 31)	*p*-value	Control (*n* = 29)	*p*-value	TRE vs. control	*p*-value
BMI	0.99 (0.08,1.90) ^a^	0.033 ^b^	0.11 (−0.03,0.26) ^a^	0.125 ^b^	−0.18 (−0.96,0.59)	0.641
PSQI	1.42 (−0.06,2.90) ^a^	0.059 ^b^	1.07 (−0.54,2.68) ^a^	0.184 ^b^	0.66 (−0.55,1.87)	0.283
Subjective sleep quality	0.00 (0.00,2.00) ^c^	0.723 ^d^	0.50 (1.00,2.00) ^c^	0.651^d^	−0.297 (−0.65,0.06)	0.100
Sleep latency	0.00 (1.00,2.00) ^c^	0.828 ^d^	0.00 (1.00,2.00) ^c^	0.169 ^d^	0.44 (−0.12,0.76)	0.007
Sleep duration	0.00 (0.00,2.00) ^c^	0.484 ^d^	0.00 (0.00,1.00) ^c^	0.335 ^d^	0.54 (0.19,0.89)	0.003
Sleep efficiency	0.00 (0.00,0.00) ^c^	0.030 ^d^	0.00 (0.00,2.00) ^c^	0.708 ^d^	−0.55 (−0.88, −0.22)	0.001
Sleep disturbances	0.42 (−0.03,0.87) ^a^	0.068 ^b^	0.50 (1.00,2.00) ^c^	0.268 ^d^	0.10 (−0.22,0.43)	0.529
Sleep medication use	0.00 (0.00,1.00) ^c^	0368 ^d^	0.00 (0.00,0.00) ^c^	0.603 ^d^	0.20 (−0.08,0.47)	0.162
Daytime dysfunction	1.00 (1.00,2.00) ^c^	0.048 ^d^	0.00 (0.00,2.00) ^c^	0.157 ^d^	0.23 (−0.12,0.57)	0.196
SBQ	0.00 (3.00,5.00) ^c^	0.189 ^d^	0.00 (3.00,5.00) ^c^	0.696 ^d^	−0.27 (−0.77,0.24)	0.293
ESS	1.84 (−0.72,4.40) ^a^	0.153 ^b^	0.38 (−1.55,2.31) ^a^	0.691 ^b^	1.49 (−0.07,3.05)	0.060
SAQLI	−0.58 (−1.14, −0.03) ^a^	0.040 ^b^	−0.02 (−0.49,0.46) ^a^	0.947 ^b^	0.36 (0.04,0.68)	0.027
Daily functioning	−0.73 (−1.56,0.11) ^a^	0.085 ^b^	0.19 (−0.58,0.96) ^a^	0.616 ^b^	0.47 (−0.01,0.96)	0.055
Social interactions	−0.90 (−1.61, −0.19)	0.015 ^b^	−0.10 (−0.79,0.59) ^a^	0.768 ^b^	0.41 (−0.07,0.89)	0.091
Emotional functioning	−0.40 (−1.14,0.34) ^a^	0.276 ^b^	−0.25 (−0.85,0.35) ^a^	0.402 ^b^	0.58 (0.16,1.00)	0.008
Symptoms	−0.10 (−0.29,0.09) ^a^	0.279 ^b^	0.10 (−0.49,0.46) ^a^	0.947 ^b^	0.01 (−0.11,0.14)	0.831

CI, confidence interval; TRE, time-restricted eating group; BMI, body mass index; PSQI, Pittsburgh Sleep Quality Index; SBQ, STOP-Bang questionnaire; ESS, Epworth Sleeping Scale; SAQLI, Calgary Sleep Apnea Quality of Life Index. ^a^ mean difference (95% confidence interval), ^b^ paired sample *t*-test; ^c^ median difference (interquartile range); ^d^ Wilcoxon signed-rank test; between-group comparisons were derived from a linear mixed model adjusted for baseline values of the outcome, age, AHI, gender, education level, employments status, marital status, and family monthly income.

Given this heterogeneity in responses, exploratory analyses were conducted. When participants were categorized by a clinically meaningful BMI change threshold (±0.5 kg/m^2^), the distribution of weight loss, stable, and weight gain categories differed significantly between groups (*p* = 0.001). Weight gain was more frequent in the TRE group (29.0%) than in the control group (10.3%) (odds ratio: 2.81, 95% CI: 0.84 to 9.36; *p* = 0.071). Furthermore, within the TRE group, both higher baseline BMI and greater adherence were associated with weight loss (*p* = 0.010 and *p* < 0.001, respectively).

### 3.3 Sleep quality

**Figure 3:**
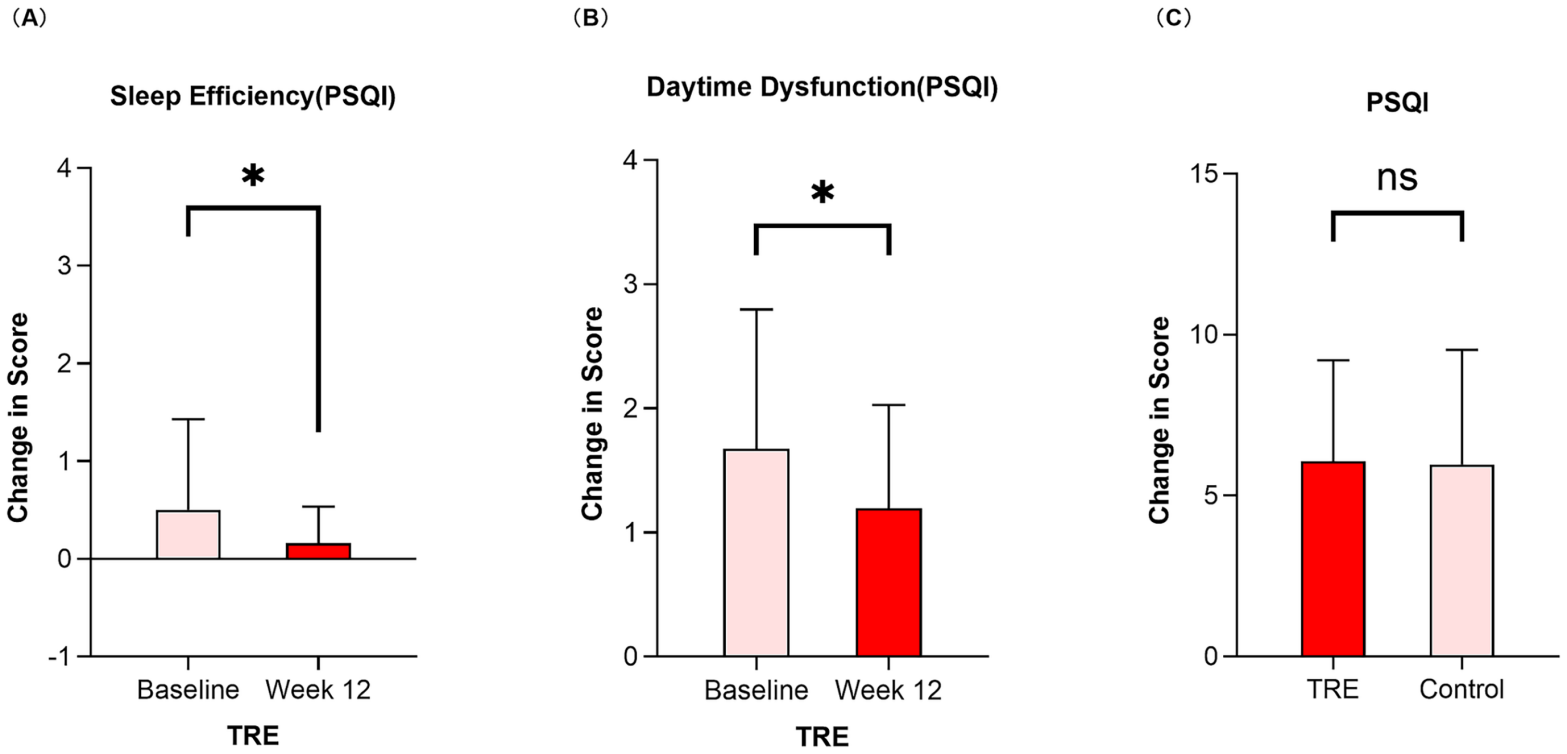
Change in sleep efficiency and daytime dysfunction in the TRE after 12 weeks and comparison of PSQI total score between TRE and control group by week 12. TRE, time-restricted eating group. PSQI, Pittsburgh Sleep Quality Index. **(A)** Change in sleep efficiency (measured by the PSQI) in the TRE from baseline to week 12. Wilcoxon signed-rank test was used. **(B)** Change in daytime dysfunction (measured by the PSQI) in the TRE from baseline to week 12. Wilcoxon signed-rank test was used. **(C)** Comparison of PSQI total score between TRE and control group by week 12. Linear mixed model was used.

Regarding sleep quality, following the 12-week intervention, intra-group analysis showed that the TRE group exhibited significant improvements in the sleep efficiency and daytime dysfunction components of the PSQI compared to baseline (*p* = 0.030 and *p* = 0.048, respectively; Figures 3A,B). In contrast, no significant changes in PSQI component or total scores were observed within the control group from baseline (Table 3). At 12 weeks, the linear mixed model analysis indicated that the intervention effect (group-by-time interaction) was not statistically significant for the PSQI total score (adjusted MD: 0.66, 95% CI: −0.55 to 1.87; *p* = 0.283; Figure 3C). However, exploratory analysis of individual PSQI components suggested specific benefits of TRE. At week 12, compared with the control group, the TRE group demonstrated significantly greater improvement in sleep latency (adjusted MD: 0.44, 95% CI: 0.12 to 0.76; *p* = 0.007), sleep duration (adjusted MD: 0.54, 95% CI: 0.19 to 0.89; *p* = 0.003), and sleep efficiency (adjusted MD: -0.55, 95% CI: −0.88 to −0.22; *p* = 0.001). No significant between-group differences were observed for the remaining components (Table 3).

### 3.4 SBQ score and excessive daytime sleepiness

Linear mixed model analysis showed no significant intervention effects on either the SBQ score or excessive daytime sleepiness (assessed by the ESS). For the SBQ score, the group-by-time interaction was not significant [F (1, 128) = 1.514, *p* = 0.221], with an adjusted mean difference of −0.27 at 12 weeks (95% CI: −0.77 to 0.24; *p* = 0.293). Similarly, for the ESS, the group-by-time interaction was not significant [*F* (1, 128) = 0.748; *p* = 0.389], and the adjusted mean difference was 1.49 (95% CI: −0.07 to 3.05; *p* = 0.060) (Table 3). Changes from baseline within each group were also not statistically significant (Table 3).

### 3.5 Quality of life

**Figure 4:**
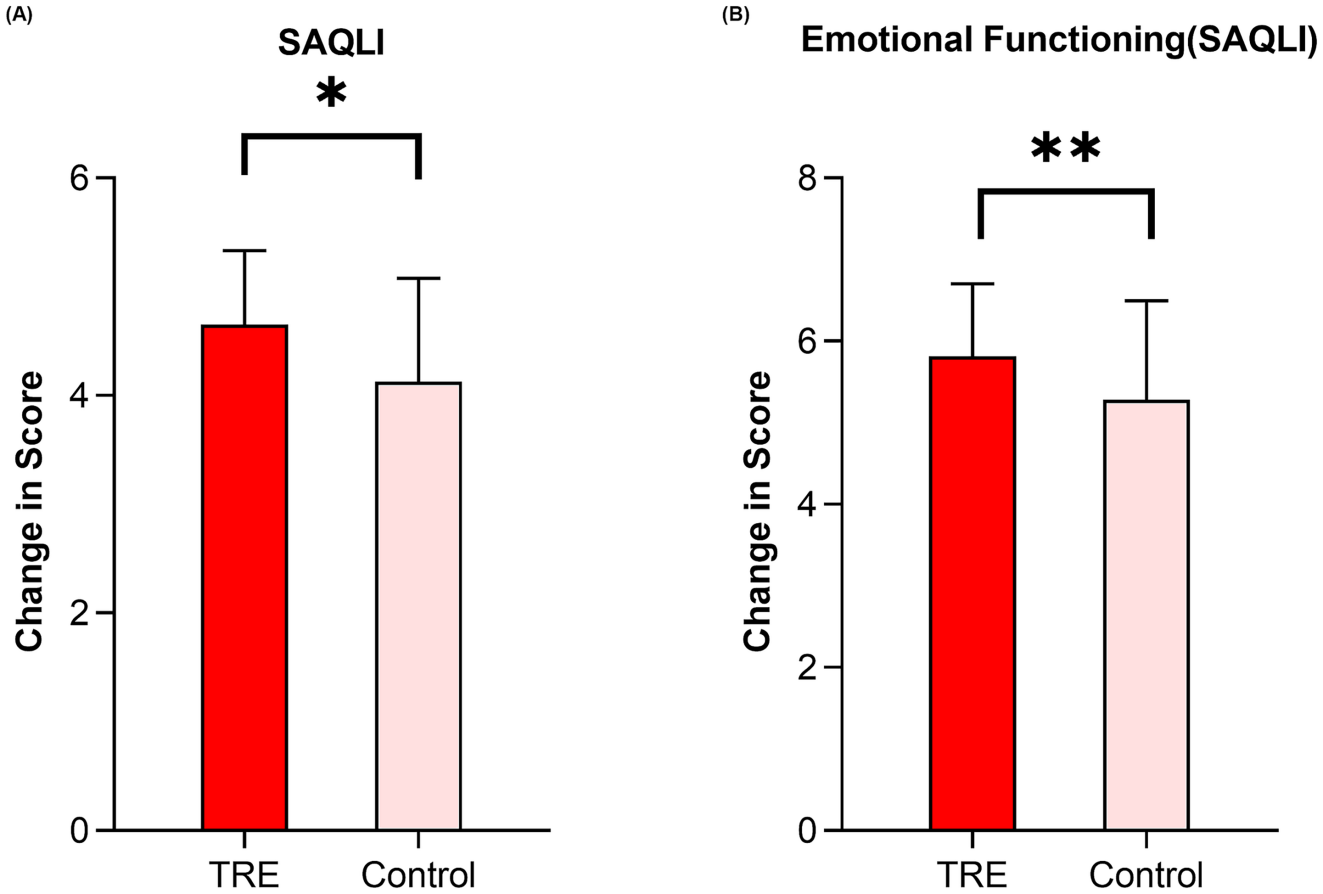
Change in emotional functioning and the total SAQLI score between TRE and control group by week 12. TRE, time-restricted eating group. SAQLI, Calgary Sleep Apnea Quality of Life Index. **(A)** Change in the PSQI total score between TRE and control group by week 12. Linear mixed model was used. **(B)** Change in emotional functioning (measured by the SAQLI) between groups. Linear mixed model was used.

Quality of life was evaluated using the SAQLI questionnaire. For the total SAQLI score, the interaction between group and time was not statistically significant [F (1,128) = 3.082; *p* = 0.082]. However, at week 12, the TRE group had a significantly higher total score than the control group (adjusted MD: 0.36, 95% CI: 0.04 to 0.68; *p* = 0.027; Figure 4A). Exploratory analysis of the SAQLI components showed distinct patterns. The TRE group showed greater improvement than the control group at 12 weeks in emotional functioning (adjusted MD: 0.58, 95% CI: 0.16 to 1.00; *p* = 0.008; Figure 4B). In contrast, no significant between-group differences were observed for the daily functioning (adjusted MD: 0.47, 95% CI: −0.01 to 0.96; *p* = 0.055), social interactions (adjusted MD: 0.41, 95% CI: −0.07 to 0.89; *p* = 0.091), and symptoms (adjusted MD: 0.01, 95% CI: −0.11 to 0.14; *p* = 0.831) (Table 3). In within-group analyses, the TRE group showed significant improvements from baseline in the total SAQLI score and the social interactions component (*p* = 0.040 and *p* = 0.015, respectively; Table 3). In contrast, the control group did not show significant changes from baseline in any SAQLI component or the total score (see Table 3).

## 4 Discussion

This randomized controlled trial is among the first to evaluate the efficacy of a theory-informed, 12-week 8-h time-restricted eating intervention in obese patients with Obstructive Sleep Apnea-hypopnea Syndrome. Our findings indicate that TRE, systematically developed using the Behavior Change Wheel framework, effectively induced weight loss and yielded significant improvements in specific domains of sleep and quality of life compared to standard care. However, its effects on global sleep quality scores, OSAHS risk perception, and subjective daytime sleepiness were not superior to the control group, underscoring the nuanced nature of dietary interventions in this complex patient population.

The significant weight loss observed within the TRE group aligns with the growing body of evidence on TRE’s efficacy (41, 55). The structured eating window, guided by BCW-derived behavior change techniques (e.g., self-monitoring, social support), likely facilitated better adherence, as reflected in the mean adherence rate of 74.8% to the prescribed eating window, and reduced unstructured snacking (37, 56). Physiologically, the imposed daily fasting period may have promoted lipolysis and improved metabolic flexibility (57–59). The lack of a statistically significant between-group difference, despite a clinically meaningful 3.2% BMI reduction in the TRE group, should be interpreted with caution. A post-hoc power analysis revealed our study was underpowered (approx. 58%) to detect a modest effect size, a limitation likely attributable to the COVID-19 recruitment constraints. Thus, the potential superiority of TRE over standard care for weight loss in this population warrants verification in larger, adequately powered trials.

The good compliance observed in this study (mean adherence rate 74.8%) supports the feasibility and acceptability of time-restricted eating among OSAHS patients with obesity. The pragmatic design adopted in this study specified an eating window without caloric prescription, thereby prioritizing ecological validity to reflect real-world application. This design choice likely underpins the heterogeneous weight responses observed, where weight gain in some participants may indicate compensatory energy intake during the permitted eating period, while significant loss in others correlated with higher baseline BMI and stricter adherence. Consequently, while supportive of TRE’s clinical utility for weight management, these findings do not establish the relative contributions of concomitant caloric restriction versus fasting-induced metabolic adaptations. Future mechanistic studies employing isocaloric designs are required to isolate the specific physiological effects of TRE.

The effects of TRE on sleep outcomes were multidimensional. The global PSQI score did not show a significant between-group difference. This finding is consistent with several recent meta-analyses of TRE (60, 61), and aligns specifically with the 2021 trial by Cienfuegos et al. (55), which also reported no improvement in PSQI among adults with obesity after an 8-week TRE intervention. This commonality suggests that generic sleep quality tools may be limited in assessing TRE’s impact. However, critical design differences between the studies illuminate a more nuanced picture. Cienfuegos et al. (55) investigated shorter (4-h or 6-h) TRE in a general obese population without diagnosed OSAHS, whereas our 12-week trial evaluated an 8-h TRE protocol specifically in patients with comorbid obesity and OSAHS. This longer intervention duration and the focus on a clinical population with pronounced sleep pathophysiology may explain the between-group benefits in sleep latency, duration, and efficiency, as well as the intra-group improvements in domains such as sleep efficiency and daytime dysfunction, observed in our study but not in the prior one. The 8-h window likely contributed to the good adherence observed in this study (mean 74.8%), which may have been a factor in facilitating the specific sleep benefits we identified. This discrepancy underscores that TRE’s effects on sleep may be contingent upon intervention characteristics and patient phenotype. The significant enhancements in quality of life, particularly in higher total SAQLI score and emotional functioning at week 12 than the control group, are noteworthy. A positive within-group change in social interactions was also observed. These improvements likely reflect a synergy between weight loss, reduced OSAHS symptom burden, and the psychosocial benefits of participating in a structured intervention (62).

The absence of significant changes in the STOP-Bang questionnaire and Epworth Sleepiness Scale scores highlights an important distinction. TRE likely exerts its benefits through systemic metabolic improvements and general symptom alleviation rather than directly modifying the anatomical risk factors for airway collapse captured by the SBQ (63). Furthermore, the ESS, while validated, may be insufficiently sensitive to detect subtle changes in daytime sleepiness in intervention studies. Future research would benefit from incorporating objective measures of OSAHS severity, such as home sleep apnea testing, to more precisely quantify TRE’s impact on the underlying pathophysiology.

Several limitations must be acknowledged. First, the single-center design and the final sample size, which was smaller than originally planned, resulted in limited statistical power and may increase the risk of a type II error, thereby constraining the generalizability of our findings. Second, the 12-week duration precludes assessment of the long-term sustainability and safety of TRE in this population; future studies with 6- and 12-month follow-ups are essential. Third, the reliance on self-reported dietary adherence and outcome measures (sleep, QoL) introduces potential for bias; future work should incorporate objective biomarkers and actigraphy. Fourth, due to the pragmatic trial design, detailed dietary intake was not strictly monitored. This precludes a definitive analysis of the extent to which the observed weight loss was attributable to a calorie deficit. Finally, while the BCW framework is a strength, the lack of a formal process evaluation limits our understanding of which specific intervention components were most active. Despite these limitations, our study provides valuable preliminary evidence for TRE’s role in managing OSAHS and obesity.

In conclusion, this theory-driven study demonstrates that time-restricted eating is a feasible and promising adjuvant intervention for weight management and improving specific patient-centered outcomes in obese individuals with OSAHS. While TRE is unlikely to serve as a monotherapy, it constitutes a valuable component of a multimodal management strategy. Future large-scale, long-term trials are warranted to confirm its efficacy, elucidate its mechanisms, and define its optimal place within integrated care models for this prevalent comorbidity.

## 5 Conclusion

This study provides evidence that a theory-informed, 12-week 8-h TRE intervention is an effective strategy for inducing weight loss and improving specific aspects of sleep (efficiency, latency, and daytime dysfunction) and quality of life in obese patients with OSAHS. While its effects on global sleep quality scores, OSAHS risk perception, and daytime sleepiness were not superior to standard care in this potentially underpowered trial, TRE represents a well-tolerated and promising dietary adjunct to standard OSAHS management, particularly for weight control. Integrating TRE into a comprehensive care model for OSAHS and obesity warrants further investigation in larger, longer-term trials that incorporate objective outcome measures.

## Data Availability

The original contributions presented in the study are included in the article/supplementary material, further inquiries can be directed to the corresponding authors.
